# Zebrafish *nampt-a* mutants are viable despite perturbed primitive hematopoiesis

**DOI:** 10.1186/s41065-024-00318-y

**Published:** 2024-04-29

**Authors:** Autumn Penecilla Pomreinke, Patrick Müller

**Affiliations:** 1 Friedrich Miescher Laboratory of the Max Planck Society, Tübingen, Germany; 2 University of Hohenheim, Stuttgart, Germany; 3 University of Konstanz, Konstanz, Germany

## Abstract

**Background:**

Nicotinamide phosphoribosyltransferase (Nampt) is required for recycling NAD^+^ in numerous cellular contexts. Morpholino-based knockdown of zebrafish *nampt-a* has been shown to cause abnormal development and defective hematopoiesis concomitant with decreased NAD^+^ levels. However, surprisingly, *nampt-a* mutant zebrafish were recently found to be viable, suggesting a discrepancy between the phenotypes in knockdown and knockout conditions. Here, we address this discrepancy by directly comparing loss-of-function approaches that result in identical defective transcripts in morphants and mutants.

**Results:**

Using CRISPR/Cas9-mediated mutagenesis, we generated *nampt-a* mutant lines that carry the same mis-spliced mRNA as *nampt-a* morphants. Despite reduced NAD^+^ levels and perturbed expression of specific blood markers, *nampt-a* mutants did not display obvious developmental defects and were found to be viable. In contrast, injection of *nampt-a* morpholinos into wild-type or mutant *nampt-a* embryos caused aberrant phenotypes. Moreover, *nampt-a* morpholinos caused additional reduction of blood-related markers in *nampt-a* mutants, suggesting that the defects observed in *nampt-a* morphants can be partially attributed to off-target effects of the morpholinos.

**Conclusions:**

Our findings show that zebrafish *nampt-a* mutants are viable despite reduced NAD^+^ levels and a perturbed hematopoietic gene expression program, indicating strong robustness of primitive hematopoiesis during early embryogenesis.

**Supplementary Information:**

The online version contains supplementary material available at 10.1186/s41065-024-00318-y.

## Introduction

Hematopoiesis is essential for the development and homeostasis of all vertebrates [18, 21]. Blood cell development occurs in two waves – primitive and definitive hematopoiesis – and the underlying gene regulatory networks are evolutionarily conserved [6, 10, 11, 15]. It has recently been reported that balanced NAD^+^ levels are essential for hematopoiesis in humans, mice and zebrafish [31]. NAD^+^ levels are balanced by nicotinamide phosphoribosyltransferase (Nampt), the rate-limiting enzyme of the salvage pathway to recycle NAD^+^ [47] (Supplementary Fig. 1A). While NAD^+^ has functions in numerous cellular processes from metabolism to DNA repair [4, 5, 19, 20], in the context of hematopoiesis the role of NAD^+^ as a co-factor for Sirtuin protein deacetylases is thought to be important [31]. Specifically, NAD^+^-dependent Sirtuin-2 has been shown to deacetylate Lmo2, a key transcription factor required to establish the blood cell lineage [6, 16, 29, 31, 36]. During primitive hematopoiesis, Lmo2 and Scl form a complex to convert lateral plate mesoderm into hemangioblast cells expressing the blood transcription marker *gata2a* and the vascular endothelial receptor *kdrl*, which is indicative of hemangioblast bipotency [6, 16] (Supplementary Fig. 1B). Lmo2 has been suggested to undergo a conformational change upon deacetylation, allowing the docking of Scl and Gata1/2, which together form a transcriptional complex to induce blood-related cell fate programs [31] (Supplementary Fig. 1A). The expression of *gata1a* concurrent with the downregulation of *kdrl* expression in a subset of *scl*^+^ cells converts hemangioblast to blood progenitor cells [15, 36], but in the absence of *gata1a* the hemangioblast differentiates into endothelial cells [16] (Supplementary Fig. 1B).

In zebrafish embryos, morpholino-mediated knockdown of *nampt-a* caused developmental defects and a decrease of lateral plate mesoderm-derived cells (*draculin:GFP*-positive) in the blood island, a major site for primitive hematopoiesis [6, 13, 31]. The decrease in the number of *draculin:GFP* cells in *nampt-a* morphants could be rescued by overexpressing a deacetylation-mimicking mutant Lmo2 protein, supporting the idea that posttranslationally modified Lmo2 acts downstream of Nampt-a and Sirtuin-2 [31]. In agreement with a primitive hematopoiesis defect, it was shown that the progression of hematopoiesis can be altered by modulating Nampt. Lower NAD^+^ levels in *nampt-a* morphants led to reduced expression of the erythroid lineage markers *gata1a* and *klf1* as well as the hemangioblast/endothelial marker *kdrl*, the latter of which could be rescued by overexpression of deacetylation-mimicking mutant Lmo2 [31]. These results suggest that hematopoiesis can be stalled by blocking NAD^+^-dependent Sirtuin-2 activity through reduced Nampt-a levels.

However, a later study showed that *nampt-a* mutants are viable [38] unlike *nampt-a* morphants, which are malformed and die during embryogenesis [13, 31]. The *nampt-a* mutants described by Ratnayake et al. [38] carry an indel in exon 2, which leads to three altered amino acids followed by a premature stop codon. In these zebrafish mutant embryos, the mutant transcript was reduced compared to the wild-type mRNA, suggesting nonsense-mediated decay [12]. It has been speculated that in zebrafish *nampt-a* mutants, the homolog Nampt-b could provide sufficient NAD^+^ [38], but it is currently unclear whether this is achieved by upregulation of Nampt-b to genetically compensate for *nampt-a* loss-of-function. The inconsistency in the phenotypes of *nampt-a* morphants [13, 31] and *nampt-a* mutants [38] leaves open the question whether Nampt-a is essential for zebrafish hematopoiesis and viability. This reflects a wider problem in the often-disparate correlations between phenotypes of mutants and knockdown approaches in multiple model organisms [7, 14, 23, 41, 46]. It is conceivable that *nampt-a* morpholinos may have a toxic off-target effect [43] and that the observed phenotypes are unrelated to *nampt-a* knockdown. On the other hand, the currently available *nampt-a* mutants [38] have a nonsense mutation that may lead to nonsense-mediated decay and an upregulation of its homolog Nampt-b by genetic compensation [12, 27, 28] to buffer fluctuations in NAD^+^ levels.

Here, we re-visit the role of Nampt-a in zebrafish hematopoiesis by directly addressing the discrepancy in the phenotypes resulting from *nampt-a* knockdown and knockout approaches. We generated CRISPR/Cas9-induced *nampt-a* mutants that recapitulate a morpholino-mediated mis-splicing effect. Despite reduced NAD^+^ levels, maternal-zygotic *nampt-a* mutants were viable and fertile. While both mutants and morphants carry identical mis-spliced mRNA containing a premature stop codon, the expression of the *nampt-a* homolog *nampt-b* did not significantly change in either loss-of-function approach compared to wild-type embryos. *draculin:GFP* cell populations were comparable in wild-type and *nampt-a* mutant embryos, suggesting that the lateral plate mesoderm, from which hematopoietic cells emerge, is not affected in the absence of Nampt-a. Both *nampt-a* loss-of-function approaches caused a decrease in the expression of the erythroid maturation marker *klf1* and the endothelial receptor *kdrl*, but not in the expression of the erythroid specification gene *gata1a*, confirming the observations in morphants that specific blood-related genes are affected in the absence of Nampt-a. Our findings show that zebrafish embryos are viable despite altered NAD^+^ levels and changes in the expression of key hematopoietic transcription factors, pointing to remarkably robust mechanisms acting during early development.

## Results

### Generation of *nampt-a* mutants

**Fig. 1:**
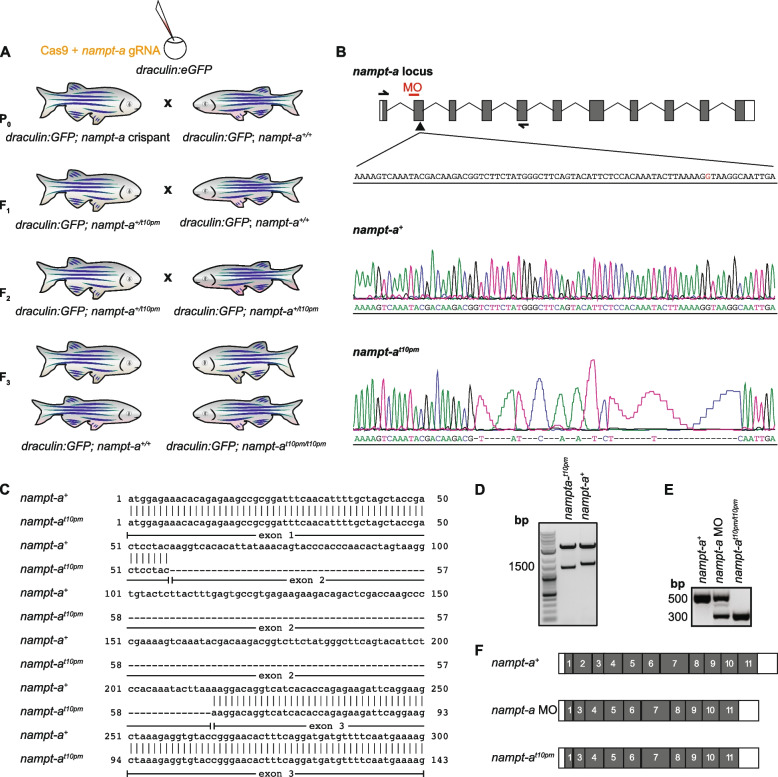
CRISPR/Cas9-mediated generation of *nampt-a* mutants for comparison to morpholino-induced mis-spliced transcripts. **A** To generate homozygous *nampt-a*^*t10pm*^ mutants using CRISPR/Cas9, *draculin:GFP* embryos were injected with Cas9 and gRNA targeting exon 2 of the *nampt-a* gene. P_0_ *nampt-a* crispants were backcrossed to *draculin:GFP* fish, and heterozygous mutant progeny was raised to adulthood (F_1_). Of these fish, the *nampt-a*^*t10pm*^ mutant allele was identified to have exon 2 absent in the mature transcript. F_1_ fish were backcrossed to *draculin:GFP* to further eliminate potential off-target mutations. F_2_ fish were in-crossed to establish homozygous *nampt-a*^*t10pm*^ mutants (F_3_). F_3_ progeny homozygous for the wild-type allele *nampt-a*^+^ were also raised to serve as controls for the genetic background. **B** Schematic of the *nampt-a* locus. Boxes represent exons, white bars represent UTRs, and thin black lines represent introns. The arrowhead marks the gRNA target site for CRISPR/Cas9-mediated mutagenesis. The red bar indicates the morpholino (MO) binding site at the 3’ splice site of the intron 1 and exon 2 splice junction on *nampt-a* pre-mRNA. Half-headed arrows represent the primer-annealing sites used to amplify and sequence *nampt-a* mature mRNA from exon 1 to exon 5. The inlet represents a short segment of the exon 2 sense strand including the 5’ splice site ‘G’ in red between exon 2 and intron 2. Lower panels show Sanger sequences of wild-type *nampt-a* and the *nampt-a*^*t10pm*^ allele with a 47 bp indel. **C** Alignment of wild-type *nampt-a*^+^ and *nampt-a*^*t10pm*^ cDNA shows that exon 2 is absent in *nampt-a*^*t10pm*^ mutant transcripts. **D** Cloning of wild-type *nampt-a*^+^ and *nampt-a*^*t10pm*^ into the *pCS2(* +*)* vector with subsequent release of inserts by restriction digestion shows that the *nampt-a*^*t10pm*^ allele is shorter than the wild-type cDNA. **E** PCR products amplified from wild-type *nampt-a*, *nampt-a* morphants (*nampt-a MO*), and *nampt-a*^*t10pm/t10pm*^ cDNA using primers shown in (**B**) indicate similar splicing defects in morphants and mutants, albeit substantial wild-type mRNA is still present in the morphants. **F** Schematic of *nampt-a* wild-type mature mRNA aligned to *nampt-a* morphant and *nampt-a*^*t10pm*^ mutant mature mRNA. Exons are shown in gray and UTRs in white boxes. Exon 2 is spliced out in both loss-of-function approaches

To compare loss-of-function phenotypes induced by morpholino-mediated knockdown and knockout, we aimed to generate zebrafish *nampt-a* mutants (Fig. 1A) that mimic the exon-skipping effect of previous morpholinos [31]. The splice-site *nampt-a* morpholino binds at the 5’ end of exon 2 in the *nampt-a* mRNA causing exon 2 to be spliced out (Fig. 1B) [31]. We therefore designed and injected gRNAs targeting the *nampt-a* gene locus at exon 2 and screened for progeny lacking exon 2 in the *nampt-a* transcript. The resulting homozygous *nampt-a*^*t10pm*^ mutant line had a 47 bp deletion spanning the 3’ end of exon 2 and the 5’ end of intron 2, thus deleting the 5’ splice-site consensus sequence (Fig. 1B). Interestingly, while the deletion of the 5’ splice-site of intron 2 should cause splicing-in of intron 2, the resulting full-length *nampt-a*^*t10pm*^ transcript lacked exon 2 and was therefore shorter than the wild-type transcript (Fig. 1C) – similar to the *nampt-a* morphant transcript (Fig. 1D-F). The mutation may have deleted exon-splicing enhancers [42], resulting in the spliceosome not recognizing exon 2. Therefore, *nampt-a* morphants and *nampt-a*^*t10pm*^ mutants have the same mature mRNA, except that *nampt-a* morphants still retained a substantial amount of wild-type *nampt-a* mRNA (Fig. 1E).

### *nampt-a* mutants have normal gross morphology in contrast to *nampt-a* morphants

**Fig. 2:**
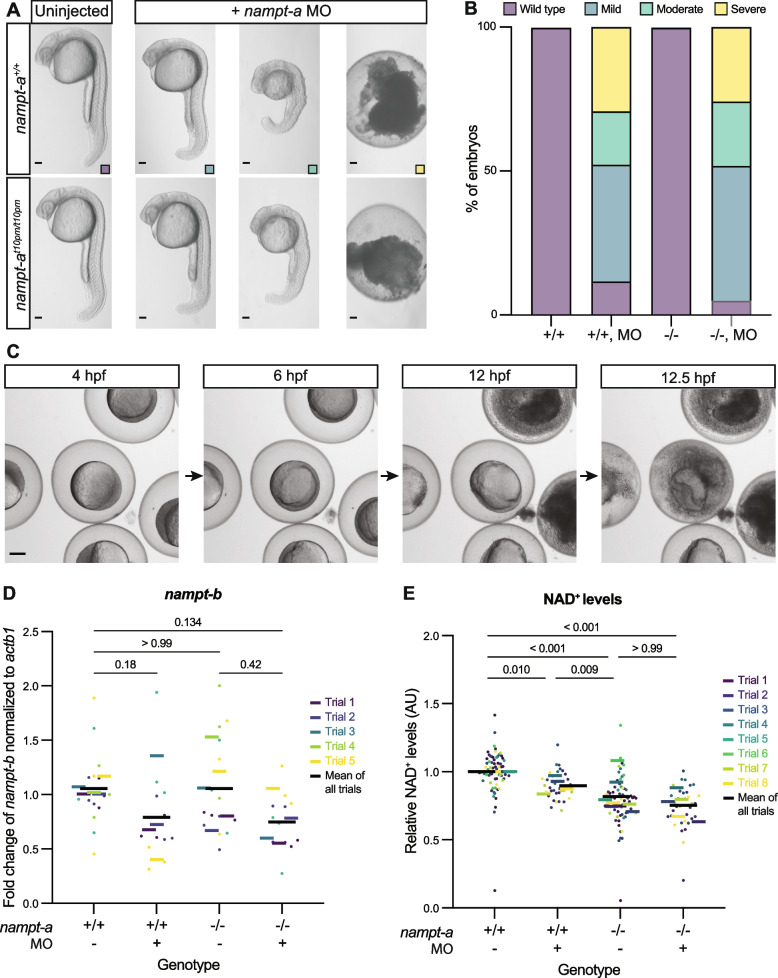
The *nampt-a* morpholino has off-target effects that lead to the maldevelopment of wild-type and mutant *nampt-a* embryos. **A** Phenotypes of *nampt-a*^+*/*+^ and *nampt-a*^*t10pm/t10pm*^ embryos injected with *nampt-a* morpholino (*nampt-a* MO). Scale bars represent 100 µm. **B** Phenotype distributions of *nampt-a*^+*/*+^ (+ / +) and *nampt-a*^*t10pm/t10pm*^ (-/-) embryos injected with *nampt-a* MO. Results are a combination of four experimental trials. + / + , *n* = 366; + / + with MO, *n* = 232; -/-, *n* = 351, and -/- with MO, *n* = 201. **C** Time-lapse imaging of embryos with severe phenotypes injected with *nampt-a* MO at 4, 6, 12 and 12.5 h post-fertilization. Scale bar represents 100 µm. **D** Wild-type *nampt-a*^+*/*+^ (+ / +) and mutant *nampt-a*^*t10pm/t10pm*^ (-/-) embryos were injected with *nampt-a* MO. Embryos were assayed at 1 dpf for *nampt-b* expression by qRT-PCR, and fold change in expression was calculated using the ∆∆C_t_ method [26]. Each point represents one biological sample of ten embryos pooled for RNA extraction. Each colored line represents the average from separate experimental trial days, and the black line shows the average from all trials. For statistical analysis in (**D**, **E**), the Kruskal–Wallis non-parametric test was applied followed by Dunn’s multiple comparison test. **E** Each point represents NAD^+^ levels in individuals relative to uninjected *nampt-a*^+*/*+^ embryos. Each colored line represents the average from separate experimental trial days, and the black line shows the average from all trials

To determine whether the aberrant morphology of *nampt-a* morphants is related to loss of *nampt-a* function, we compared the phenotypes resulting from the two loss-of-function approaches. Homozygous *nampt-a*^*t10pm/t10pm*^ embryos at 1 day post-fertilization (dpf) appeared similar to *nampt-a*^+*/*+^ wild-type cousin controls (Fig. 2A) and became fertile adults. This is consistent with the *nampt-a* mutant reported by Ratnayake et al. [38]. In contrast, morpholino-injected *nampt-a*^*t10pm/t10pm*^ embryos and *nampt-a*^+*/*+^ cousin controls exhibited a range of malformations (Fig. 2A, B). Mild morphant phenotypes included smaller eyes and heads, enlarged inner vacuolated cells in the notochord, damaged yolk extensions and disorganized somites that lacked the typically well-defined chevron shape observed in uninjected siblings. In moderate phenotypes, further reduction in head size and curved spinal structures led to a shortening of the body axis. In severe cases, embryos first exhibited substantial yolk leakage, then lost integrity and disintegrated as the yolk burst before completion of epiboly (Fig. 2C). Since *nampt-a*^*t10pm/t10pm*^ and *nampt-a*^+*/*+^ were similarly affected, regardless of their genetic background (Fig. 2), this suggests that the aberrant *nampt-a* morphant phenotype is caused by an off-target effect of the morpholino.

Premature stop codons in the mature mRNA are recognized by the nonsense-mediated decay pathway and have been reported to trigger transcriptional adaptation, leading to the upregulation of homologous genes to buffer against deleterious mutations [12, 28]. Since *nampt-a*^*t10pm*^ mutant mRNA carries a premature stop codon (Supplementary Note, Supplementary Fig. 2A, B), we investigated whether the morphological wild-type appearance of *nampt-a*^*t10pm/t10pm*^ mutants might be due to genetic compensation by Nampt-b. We used qRT-PCR to quantify *nampt-b* expression in uninjected *nampt-a*^+*/*+^ and *nampt-a*^*t10pm/t10pm*^ embryos as well as morpholino-injected siblings from both lines (Fig. 2D). There was no significant difference between *nampt-b* transcript levels in *nampt-a*^+*/*+^ and *nampt-a*^*t10pm/t10pm*^ (Fig. 2D, mean difference (∆) = 0.001, *p* > 0.99). This suggests that *nampt-a* loss-of-function in mutants is not genetically compensated by upregulation of *nampt-b* expression.

Surprisingly, morpholino injections caused a mild reduction of *nampt-b* expression in *nampt-a*^+*/*+^ and *nampt-a*^*t10pm/t10pm*^ compared to uninjected *nampt-a*^+*/*+^ embryos (∆ = 0.26, *p* = 0.181; ∆ = 0.306, *p* = 0.134, Fig. 2D). To address whether combined downregulation of *nampt-a* and *nampt-b* might cause reduced NAD^+^ levels to explain the more severe phenotype in morphants compared to mutants, we quantified the NAD^+^ levels in different genetic backgrounds. We found that the NAD^+^ levels of *nampt-a*^+*/*+^ morphants, uninjected *nampt-a*^*t10pm/t10pm*^ embryos, and *nampt-a*^*t10pm/t10pm*^ morphants were significantly lower than the NAD^+^ levels in uninjected *nampt-a*^+*/*+^ embryos (Fig. 2E, ∆ = 0.10, *p* = 0.010; ∆ = 0.18, *p* < 0.001; ∆ = 0.25, *p* < 0.001, respectively). Additionally, NAD^+^ levels were significantly lower in uninjected *nampt-a*^*t10pm/t10pm*^ embryos compared to *nampt-a*^+*/*+^ morphants (∆ = 0.08, *p* = 0.009), suggesting a stronger NAD^+^ reduction in mutants than in morphants. This is in line with the remaining wild-type *nampt-a* mRNA in morphants (Fig. 1E). Additionally, the morpholino injections did not cause further reduction of NAD^+^ levels in *nampt-a* mutants. Therefore, lower NAD^+^ levels as predicted from a mild reduction in *nampt-b* expression in morphants cannot account for the aberrant morphant phenotype (Fig. 2A).

Together, these results provide additional support that Nampt-a is not essential for zebrafish viability, and that zebrafish survive well even with reduced NAD^+^ levels [38].

### *nampt-a* loss-of-function causes reduced expression of specific erythrocyte and endothelial markers

**Fig. 3:**
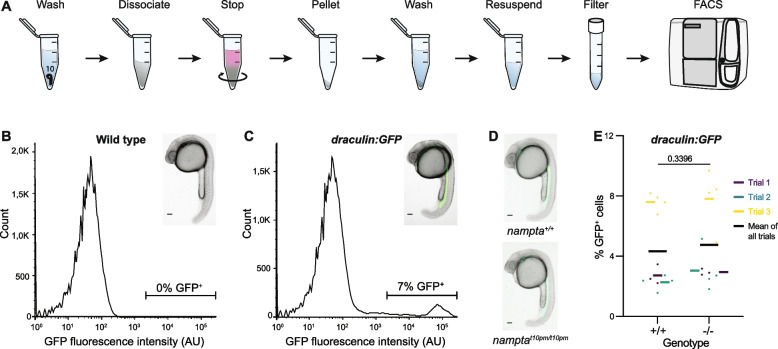
*nampt-a*^+*/*+^ and *nampt-a*^*t10pm/t10pm*^ embryos have comparable *draculin:GFP*-positive cell populations. **A** To quantify lateral plate mesoderm cells, *draculin:GFP* zebrafish embryos were homogenized, and GFP^+^ cells were counted using fluorescence activated cell sorting (FACS). **B** Wild-type embryos not expressing GFP were used as controls to establish a cut-off for GFP^−^ cells. **C** Example of FACS read-out of GFP^+^ cells (green, inset) in *draculin:GFP* embryos that are detected above the baseline cut-off set in (**B**). **D** Images of *nampt-a*^+*/*+^ and *nampt-a*^*t10pm/t10pm*^ embryos in the *draculin:GFP* background before homogenization and quantification by FACS. **E** Quantification of GFP^+^ cells in *nampt-a*^+*/*+^ (+ / +) and *nampt-a*^*t10pm/t10pm*^ (-/-) embryos. Each point represents one biological sample of ten pooled embryos. Each colored line represents the average from separate experimental trial days, and the black bar shows the mean of all trials. For statistical analysis, the Mann–Whitney test was applied to determine the *p*-value. Scale bars in **B-D** represent 100 µm

The viability of the *nampt-a* mutants suggested that hematopoiesis persists with reduced NAD^+^ levels in the absence of Nampt-a. To determine whether blood cell specification proceeds normally, we first used FACS to quantify GFP-positive (GFP^+^) cells in the *draculin:GFP* transgenic background, which marks lateral plate mesodermal cells giving rise to blood, heart, kidney and endothelial cells [32]. We optimized the cell suspension protocol from Bresciani et al. [2] and adapted it for 1 dpf zebrafish embryos (Fig. 3A, Materials and methods). To set a baseline for GFP-negative (GFP^−^) cells, embryos from non-transgenic lines were used as controls (Fig. 3B). Cells detected above the baseline represent GFP^+^ cells, and in *draculin:GFP* embryos about 7% of the measured cells were GFP^+^ (Fig. 3C). Using this protocol, we found that *nampt-a*^+*/*+^ and *nampt-a*^*t10pm/t10pm*^ embryos had similar numbers of GFP^+^ cells (Fig. 3D, E), suggesting that the lateral plate mesodermal cell population as a whole was unaffected in the absence of Nampt-a, although the assay might not be sensitive enough to detect changes in smaller sub-populations, such as specific blood cells.

**Fig. 4:**
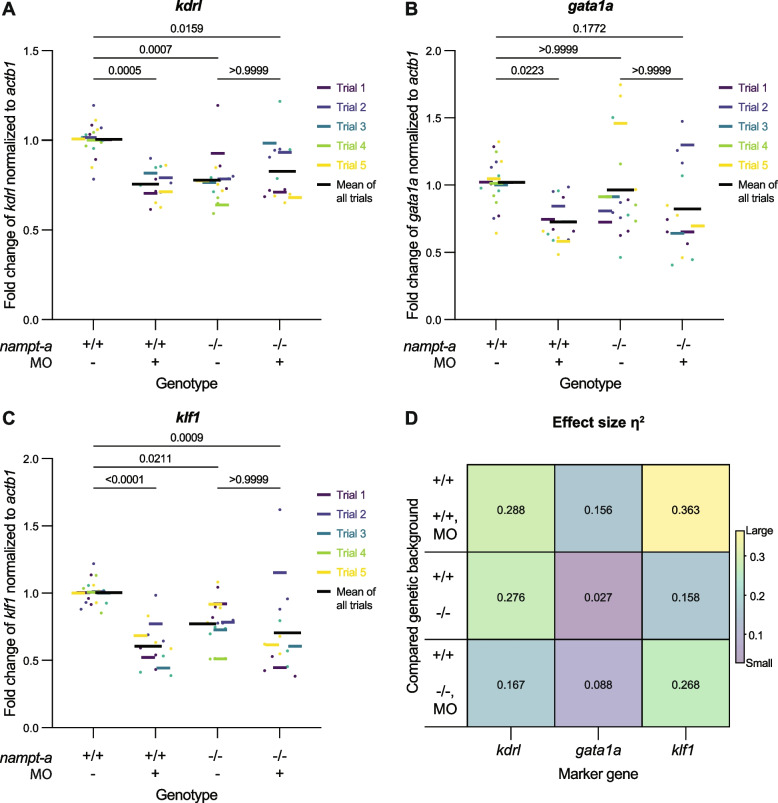
Expression of hemangioblast/endothelial and erythrocyte maturation markers in *nampt-a* loss-of-function conditions. *nampt-a*^+*/*+^ (+ / +) and *nampt-a*^*t10pm/t10pm*^ (-/-) embryos were injected with *nampt-a* morpholino (MO). Embryos were assayed at 1 dpf for the expression of the hemangioblast/endothelial marker *kdrl* (**A**) and the erythrocyte markers *gata1a* (**B**) and *klf1* (**C**) by qRT-PCR. Fold change was calculated using the ∆∆C_t_ method [26]. Each point represents one biological sample of ten embryos pooled for RNA extraction. Each colored line represents the average from separate experimental trial days with the black line representing the average from all trials. For statistical analysis, the Kruskal–Wallis non-parametric test was applied followed by Dunn’s multiple comparison test to determine *p*-values. **D** Effect size (η^2^) for the Kruskal–Wallis test to compare each group

Therefore, to assess blood differentiation markers with higher resolution we measured the expression levels of specific blood markers. We used qRT-PCR to quantify expression levels of the hemangioblast/endothelial marker *kdrl* [6, 16], the erythroid-specific transcription factor *gata1a* [40] and the erythrocyte maturation marker *klf1* [24, 34] in uninjected *nampt-a*^+*/*+^ as well as *nampt-a*^*t10pm/t10pm*^ embryos or corresponding morphants at 1 dpf (Fig. 4A-C). If Nampt-a regulates hemangioblast specification, the expression of all three genes is expected to decrease upon *nampt-a* loss-of-function. If Nampt-a has a role later in hematopoiesis – such as regulating erythrocyte commitment or maturation – *gata1a*, *klf1* or both are expected to decrease upon *nampt-a* loss-of-function.

*kdrl* expression was significantly reduced in *nampt-a*^+*/*+^ morphants (∆ = 0.25, *p* < 0.001), in uninjected *nampt-a*^*t10pm/t10pm*^ mutants (∆ = 0.23, *p* < 0.001), and in *nampt-a*^*t10pm/t10pm*^ morphants (∆ = 0.18, *p* = 0.016) compared to uninjected *nampt-a*^+*/*+^ (Fig. 4A). The effect size η^2^ [44] of the *kdrl* expression change in *nampt-a*^+*/*+^ morphants and uninjected *nampt-a*^*t10pm/t10pm*^ to uninjected *nampt-a*^+*/*+^ was similar, suggesting a reduction in hemangioblast or endothelial cells upon *nampt-a* loss-of-function (η^2^ = 0.288, η^2^ = 0.276, respectively) (Fig. 4D). Interestingly, the reduction in either hemangioblast or endothelial cells was not embryonically lethal, indicating that development can proceed with reduced NAD^+^ levels and perturbed primitive hematopoiesisis.

Compared to uninjected *nampt-a*^+*/*+^ embryos, *gata1a* expression was significantly reduced in *nampt-a*^+*/*+^ morphants (∆ = 0.30, *p* = 0.022), unaffected in uninjected *nampt-a*^*t10pm/t10pm*^ mutants (∆ = 0.06, *p* > 0.99), and mildly reduced in *nampt-a*^*t10pm/t10pm*^ morphants (∆ = 0.20, *p* = 0.117) (Fig. 4B). The effect sizes of *gata1a* expression changes in both *nampt-a*^+*/*+^ and *nampt-a*^*t10pm/t10pm*^ morphants compared to uninjected *nampt-a*^+*/*+^ (η^2^ = 0.156, η^2^ = 0.088, respectively) were greater than the effect size of uninjected *nampt-a*^*t10pm/t10pm*^ compared to uninjected *nampt-a*^+*/*+^ embryos (η^2^ = 0.027). Therefore, in contrast to the reduction in *kdrl* expression in both *nampt-a* loss-of-function approaches, *gata1a* expression was only affected in morphants, suggesting an off-target effect of the morpholino downregulating *gata1a*. Unaffected *gata1a* expression in the mutants further indicates that the commitment of hemangioblast to the erythroid cell lineage is not impaired in the absence of Nampt-a.

Finally, *klf1* expression was significantly reduced in *nampt-a*^+*/*+^ morphants (∆ = 0.40, *p* < 0.001), in uninjected *nampt-a*^*t10pm/t10pm*^ mutants (∆ = 0.23, *p* = 0.021) and in *nampt-a*^*t10pm/t10pm*^ morphants (∆ = 0.30, *p* < 0.001) compared to uninjected *nampt-a*^+*/*+^ (Fig. 4C). The decrease in *klf1* expression indicates a reduction in erythrocyte maturation upon *nampt-a* loss-of-function. The effect sizes of *klf1* expression changes in *nampt-a*^+*/*+^ and *nampt-a*^*t10pm/t10pm*^ morphants compared to uninjected *nampt-a*^+*/*+^ (η^2^ = 0.363, η^2^ = 0.268, respectively) were greater than those in uninjected *nampt-a*^*t10pm/t10pm*^ compared to uninjected *nampt-a*^+*/*+^ embryos (η^2^ = 0.158). These results suggest that the morpholino causes a further decrease in *klf1* expression compared to the mutant lines.

In summary, our findings indicate that the *nampt-a* morpholino has off-target effects on *gata1a* and *klf1* expression, but not on *kdrl*. Based on the reduction of *kdrl* upon *nampt-a* loss-of-function (Fig. 4A), Nampt-a likely supports the maintenance of hemangioblast or endothelial cell populations. The reduction of *klf1* and unaffected *gata1a* expression (Fig. 4B, C) suggest that Nampt-a may have a role in erythrocyte maturation but not in erythroid cell commitment.

## Discussion

Hematopoiesis involves an intricate interplay of signaling molecules and transcription factors to produce proper functional blood cells [6, 18]. Here, we analyzed the effects of *nampt-a* loss-of-function on embryogenesis, NAD^+^ levels and primitive hematopoiesis in zebrafish mutants and morphants with identical defective transcripts. Both nonsense *nampt-a*^*t10pm*^ mutants and splice-site morphants had reduced NAD^+^ levels, and we did not find evidence for transcriptional adaptation by upregulation of the homolog *nampt-b*. *nampt-a*^*t10pm*^ mutant lines are viable, do not display observable developmental malformations, and have similar overall lateral plate mesodermal cell populations as wild-type embryos. In contrast, injection of *nampt-a* morpholinos caused developmental defects similar to previously reported phenotypes [31] – even in *nampt-a* mutant embryos – suggesting a toxic off-target effect of the morpholino.

Discrepancies between gene knockdown with morpholinos and gene knockout in mutants have been observed in several model organisms [7, 14, 17, 45], and the correlation between phenotypes of mutants morphants is often poor [23]. This has been partly attributed to morpholino off-target effects. For example, morpholino-mediated knockdown of the *prrx1a* gene was reported to cause incorrect heart looping in zebrafish compared to control morpholinos [35]. In contrast, later studies reported that zebrafish had normal heart looping despite complete locus deletion of *prrx1a* and knockout of *prrx1* homologs [46]. Additional investigation revealed that the *prrx1a* morpholino affected heart looping-related genes that are expressed before the onset of *prrx1a*. Tessadori et al. [46] therefore concluded that the initially reported *prrx1a* morpholino phenotypes in Ocaña et al. [35] were due to morpholino off-target effects that may be upstream and independent of Prrx1 function.

Discrepancies between morphants and mutants have also been attributed to transcriptional adaptation [12], where the presence of premature termination codons in mutant mRNAs can induce the upregulation of homologous genes to buffer against deleterious mutations. According to our current understanding, transcriptional adaptation is gene- and allele-specific, requires sequence similarity of the homologous gene to the exon or intron of the mutant allele, and for some genes a sufficient amount of mutant transcript must be degraded by nonsense-mediated decay for transcriptional adaptation to occur [12, 27, 28, 45]. In contrast, complete deletion of the locus, deletion of the transcriptional start site, or translation-blocking morpholinos do not have nonsense mRNAs with premature termination codons and therefore do not activate transcriptional adaptation [12, 27, 28, 45, 46]. Whether splice-site morpholinos that cause the formation of nonsense mutant mRNAs can activate transcriptional adaptation has previously been unclear, but here we show that *nampt-b* expression was not upregulated in splice-site morphants. We also provide additional evidence that not all genes with nonsense mutations are genetically compensated by transcriptional adaptation since nonsense mutant *nampt-a*^*t10pm*^ did not induce a significant increase in the expression of the *nampt-b* homolog; however, the role of additional potential *nampt* homologs [1] still needs to be investigated.

Morphants and maternal-zygotic *nampt-a*^*t10pm/t10pm*^ mutants share defects in blood marker expression, suggesting that Nampt-a has specific roles to support erythrocyte commitment and to maintain the hemangioblast/endothelial cell population. *klf1* was reduced in *nampt-a*^*t10pm/t10pm*^ mutants and morphants, indicative of a decrease in the number of mature erythrocytes but not in erythroid stem cells. In addition, we found that *kdrl* expression was significantly reduced in either *nampt-a* loss-of-function situation. One possible explanation is that *kdrl* expression could be more dependent on the NAD^+^-dependent assembly of the Lmo2/Scl complex than other transcription factors. A reduction in hemangioblast or endothelial cell populations appears unlikely at first sight given that the expression of the erythroid marker *gata1a* is unaffected in the absence of Nampt-a. However, as a response to a potentially reduced hemangioblast cell population, hemangioblast differentiation to endothelial precursor cells may be sacrificed by downregulating *kdrl* in hemangioblasts to reach wild-type blood stem cell numbers. Previous studies have shown that hemangioblast *scl*^+^ cells downregulate *kdrl* expression and upregulate *gata1a* for blood cell commitment and vice versa for vascular endothelial commitment [8, 15, 16]. In line with this hypothesis, BMP signaling may be the switch for hemangioblast specification to either blood or endothelial cell. For example, knockdown of BMP signaling, but not other blood-related signaling pathways such as Wnt, Notch or FGF, decreased the expression of another erythrocyte differentiation marker *hba1* and increased the level of the endothelial precursor cell differentiation marker *aplnr* in *Xenopus* [33]. Whether there is a similar blood-endothelial switch in zebrafish and other organisms is an open question for future investigation. In the present study, we have only tested the effect of *nampt-a* loss-of-function on the expression of blood-related genes at one time point when blood circulation begins. It remains unclear which and how blood cells are precisely affected in the absence of Nampt-a. In future work, the *nampt-a*^*t10pm*^ mutants can be used to further dissect the Nampt-a contribution at different stages of hematopoiesis.

Surprisingly, zebrafish are viable even with strongly reduced NAD^+^ levels in the absence of Nampt-a. The viability of homozygous *nampt-a* mutants could be in line with the viability of *nampt* heterozygous mice [39]. *nampt* heterozygous mice with one functional allele are morphologically wild-type, whereas homozygous *nampt* mutant mice are embryonically lethal [39]. It is possible that the zebrafish homolog *nampt-b* [13] on its own provides NAD^+^ levels sufficient for viability [38], and in the future it will be useful to analyze *nampt-a;nampt-b* zebrafish compound mutants as well as additional *nampt* homologs [1].

## Materials and methods

### Zebrafish handling

Live embryos were kept in embryo medium containing 250 mg/l Instant Ocean sea salt mix (Aquarium Systems) and 1 mg/l methylene blue at 28°C. *draculin:GFP* [32] zebrafish embryos were collected 5 min after they were laid, and they were injected at the one-cell stage with CRISPR/Cas9 mixture containing Cas9 protein and crRNA targeting the *nampt-a* exon 2 sequence 5’-ACGACAAGACGGTCTTCTATGGG-3’ (100 mM stock concentration) (IDT). At 1 day post-fertilization (dpf), injected embryos were assayed for mutagenesis by amplifying the region of interest [30] followed by the T7 endonuclease I assay. Briefly, for PCR amplification the forward primer 5’-CTTTCATTGCAGGTCACACATT-3’ and the reverse primer 5’-GCTGGAACAAACAGTGGTGTTA-3’ were used with the following thermocycler program: an initial denaturation step at 94°C for 3 min, 35 cycles of 94°C for 30 s, 56°C for 30 s, 72°C for 30 s, and a final elongation step at 72°C for 5 min. The PCR products were then denatured and reannealed by heating at 95°C for 5 min, cooled down to 85°C at -2°C per second, and then cooled down to 25°C at -0.1°C per second until 4°C. The PCR product solutions were split into two tubes. One half was treated with T7 endonuclease in NEBuffer™ 2 (New England Biolabs, Cat. No. M0302), and the other half was only treated with the buffer. PCR amplicons were loaded onto 2% agarose gels for electrophoretic separation.

To identify lesions in the mature mRNA transcript, RNA was extracted from *nampt-a*^+*/*+^, *nampt-a*^*t10pm/t10pm*^ and morphant embryos using TRIzol™ (Invitrogen, Cat. No. 15596018), and cDNA was synthesized using SuperScript III First-Strand Synthesis SuperMix for qRT-PCR (Thermo Fischer Scientific, Cat. No. 11752–050). Primers flanking exon 1 and exon 5 were used for PCR amplification as previously described by Morishima et al. [31] with the forward primer 5’-AGAGAAGCCGCGGATTTCAA-3’ and the reverse primer 5’-CTCCAGTCCTTCCAGGCTTC-3’.

CRISPR/Cas9-targeted embryos were raised to adulthood and screened for germline transmission of mutations by backcrossing to *draculin:GFP* zebrafish. The *nampt-a*^*t10pm*^ line was selected from heterozygous mutant F_1_ fish (Fig. 1A). To further reduce potential off-target mutations [37], F_1_ heterozygous *nampt-a*^*t10pm/*+^ fish were backcrossed to *draculin:GFP* to produce F_2_ heterozygous *nampt-a*^*t10pm/*+^ (Fig. 1A). Heterozygous F_2_ fish were in-crossed to produce F_3_ homozygous mutant *nampt-a*^*t10pm/t10pm*^ and wild-type *nampt-a*^+*/*+^. F_3_ fish and subsequent generations were used for all other experiments with *draculin:GFP*;*nampt-a*^+*/*+^ fish serving as cousin controls.

*nampt-a*^+*/*+^ and *nampt-a*^*t10pm/t10pm*^ embryos were injected with 150 µM of *nampt-a* morpholino 5’-TGTGTGACCTGCAATGAAAGAAAGA-3’ [31] in an injection volume of 1.5 nl.

### Alignment of wild-type and putative mutant Nampt-a proteins

Putative protein products of Nampt-a^t10pm^ were predicted using Expasy [9]. and aligned to the wild-type Nampt-a (XP_002661386.1) using Clustal Omega in Jalview version 2.11.2.6 [48]. The percentage identity option was used to set the color scheme.

### Live imaging of embryos

Zebrafish embryos at 1 dpf were anesthetized, mounted in 2% methylcellulose in embryo medium and imaged on an AxioZoom V16 (Zeiss) microscope with a PlanNeoFluar Z 1x/0.25 objective. For time-lapse imaging, ten embryos were placed in embryo medium without methylene blue. The embryos were imaged for 20 h every 10 min on an ACQUIFER Imaging Machine (DITABIS AG) with the settings described in Čapek et al. [3].

### Measurement of NAD^+^ levels

Individual embryos with intact chorions at 1 dpf were placed in a 1.5 ml Eppendorf tube and homogenized with a battery-operated pestle motor mixer. The homogenized tissue was rinsed off the pestle with 150 µl of 1 × PBS. To measure NAD^+^ levels, 50 µl of homogenized tissue and 50 µl of NAD^+^/NADH-Glo™ assay master mix (Promega, Cat. No. G9071), prepared following the manufacturer’s instructions, were mixed in white 96 well-plates (LUMITRAC, Cat. No. 655075) and incubated for 45 min at room temperature. Luminescence was measured using a plate reader (BioTek Gen5). NAD^+^ levels were quantified as the ratio of luminescence measured from each embryo to the average luminescence of *nampt-a*^+*/*+^ embryos for each experimental trial.

### Quantitative reverse transcription PCR (qRT-PCR)

RNA was extracted from ten pooled embryos using TRIzol™ (Invitrogen, Cat. No. 15596018) or NucleoZol (Macherey–Nagel) following the manufacturer’s instructions and homogenized with a battery-operated pestle motor mixer. If necessary, additional RNA clean-up was performed by precipitation. Briefly, 500 µl H_2_O were added, and 50 µl of 3 M sodium acetate (pH 5.3) and 10 µg of GlycoBlue™ Coprecipitant (Thermo Fisher Scientific, Cat. No. AM9516) were mixed with the RNA solution. Then, 500 µl of room temperature isopropanol was added to the solution. The samples were mixed well and incubated at room temperature for 20 min. Subsequently, the samples were centrifuged at 12,000 g at 4°C for 10 min. The RNA pellet was washed 2 times with 500 µl of ice-cold 70% ethanol. The RNA pellet was air-dried for 15 min at room temperature and at 65°C for 1 min to remove excess alcohol. The RNA pellet was finally dissolved in 15 µl of nuclease-free H_2_O.

cDNA was generated from 500 ng of RNA using SuperScript™ III First-Strand Synthesis SuperMix for qRT-PCR (Thermo Fisher Scientific, Cat No. 11752–050) following the manufacturer’s instructions. Then, 4 µl of 1:5 diluted cDNA was mixed with qRT-PCR reaction master mix (10 µl Platinum® SYBR® Green qRT-PCR SuperMix-UDG (Thermo Fisher Scientific, Cat. No. 11733046)), 5.2 µl of nuclease-free H_2_O, 0.4 µl of 10 µM forward primer and 0.4 µl of 10 µM reverse primer for the genes of interest listed in Supplementary Table 1. The qRT-PCR thermocycler was set to 50°C for 2 min, 95°C for 2 min, 40 cycles of 95°C for 15 s, 60°C for 30 s, plate read, including a final melting curve from 55°C to 95°C with increments of 0.5°C for 5 s and plate read using CFX Connect™ Real-Time System (Bio-Rad). Note that two qRT-PCR trials in Figs. 2D and 4 were performed in a double-blind setup.

To analyze differences in gene expression, the fold change of each gene normalized to *act1b* was calculated using the ∆∆C_t_ method [26].

### Quantification of draculin:GFP cells by fluorescent activated cell sorting (FACS)

To compare *draculin:GFP*^+^ cell populations, *nampt-a*^+*/*+^ and *nampt-a*^*t10pm/t10pm*^ embryos in the *draculin:GFP* background were prepared for FACS. To dissociate embryos at 1 dpf, the protocol from Bresciani et al. [2] was adapted and modified for 1 dpf (Fig. 3A). Ten dechorionated embryos were pooled, placed into 1.5 ml Eppendorf tubes and washed with 1 ml of 1 × PBS. A dissociation mix (250 µl of 0.25% trypsin–EDTA (Sigma, T4049) and 250 µl Liberase™ (1mg/ml) (Roche, Cat. No. 05466202001)) was pre-heated to 30°C and added to the embryos. Embryos were mechanically dissociated via harsh pipetting, first with a P1000 and then with a P200 pipet for 5–10 min until the solution looked homogeneous. The reaction was stopped with a mixture of 360 µl Gibco™ DMEM (Cat. No. 11960–044) and 10% FBS (Biochrom, Cat. No. S0415) that had been preheated to 30°C. Samples were centrifuged for 5 min at 700 g at room temperature, washed and resuspended with 1 ml of 1 × PBS, and centrifuged for 5 min at 700 g. The supernatant was discarded, and the pellet was resuspended in 1 ml of 1 × PBS and filtered through a cell strainer with a 30 µm mesh. GFP^+^ cells were quantified using a BD FACSMelody™ Cell sorter (BD Biosciences). Using FlowJo v10 software, the profile of live healthy cells was gated from unhealthy cells based on their internal complexity using side scatter (SSC-A) versus the measured cell size with forward scatter (FSC-A). To ensure that only singlets were measured, the live cells’ profile was further gated based on the correlation of cell height (FSC-H) versus cell area (FSC-A) using the forward scatter optical detector. To set a baseline of GFP^−^ cells, embryos from wild-type non-transgenic lines were used as negative controls.

### Data analysis

Results of NAD^+^ and qRT-PCR measurements were plotted using Prism (GraphPad Software). To calculate *p*-value and *z*-statistics, the Kruskal–Wallis non-parametric test combined with Dunn’s multiple comparison test function was used. To determine the effect size between two specific groups for the Kruskal–Wallis test, the effect size was quantified in terms of η^2^ [22]. η^2^ was determined by entering the *z*-statistic from Prism into the effect size calculator by Lenhard and Lenhard [25]. Supplementary Table 2 shows an example of the resulting *p*-value and *z*-statistics for *kdrl*. Using the ‘*z*’ test option, the *z*-value of 3.943 and the total sample size of 54 were entered, and the resulting η^2^ for the difference in *kdrl* expression between *nampt-a*^+*/*+^ and *nampt-a*^+*/*+^ morphant was 0.288 (Fig. 4D). The results were then plotted in Prism.

### Supplementary Information

**Supplementary Material 1.**

## Data Availability

The data that support the findings of this study are available from the corresponding author.
