# Supplementary material for: Zebrafish nampt-a mutants are viable despite perturbed primitive hematopoiesis
Source: Hereditas. 2024 Apr 29;161:14. doi: 10.1186/s41065-024-00318-y (PMC11057069; doi:10.1186/s41065-024-00318-y)
Supplement: Supplementary file 1 — Supplementary Material 1. [file 41065_2024_318_MOESM1_ESM.pdf]

Supplementary Material

Zebrafish *nampt-a* mutants are viable despite perturbed primitive hematopoiesis

Autumn Penecilla Pomreinke[1,2] and Patrick Müller[1,3*]

[1]Friedrich Miescher Laboratory of the Max Planck Society, Tübingen (Germany)

[2]University of Hohenheim, Stuttgart (Germany)

[3]University of Konstanz, Konstanz (Germany)

[*]Correspondence: patrick.mueller@uni-konstanz.de

Supplementary Note

To assess the potential impact of the *nampt-a^{t10pm}* mutation, mutant protein variants were predicted *in silico* using Expasy (Duvaud et al., 2021) and aligned to full-length Nampt-a (Supplementary Fig. 2). Wild-type Nampt-a contains 493 amino acid residues and has two subdomains (Supplementary Fig. 2A) (The UniProt Consortium, 2022): A domain of unknown function from N11 to I117, and a domain belonging to the Nicotinate phosphoribosyltransferase family (NAPRTase) from Y189 to G466 (Supplementary Fig. 2A,B). Translational products predicted from the three putative open reading frames (ORF) were aligned to full-length Nampt-a (Supplementary Fig. 2C). In mutants and morphants lacking exon 2, translation from the canonical start-site AUG results in a premature stop codon, and the Nampt-a mutant protein product has 96 residues, including aberrant amino acids (Supplementary Fig. 2A-C: ORF1). There are also short protein fragments predicted from alternative start codons in ORF2 and one containing the NAPRTase domain in ORF3.

Supplementary Table 1. Primers for qRT-PCR. To measure the fold change in the expression of blood-related marker genes and *nampt-b* normalized to *actb1* as a housekeeping gene, the following primer pairs were used for qRT-PCR. F: forward primer, R: reverse primer. Primers for *nampt-b* were designed by using the accession numbers as input for Primer-Blast (NCBI, 1988).

**Primer name**	**5' to 3' sequence**	**Reference**
actb1_F	GCTGTTTTCCCCTCCATTGTT	Morishima et al. (2019)
actb1_R	TCCCATGCCAACCATCACT	
gata1a_F	ACTCCTCTGAGCCTTCTCGT	
gata1a_R	GACTTGGCGAACTGGACTGT	
kdrl_F	CTGGTGGAGAGGCTAGGAGA	
kdrl_R	TGATCGGGATGTAGTGCTTTC	
klf1_F	CTCCGTACACACACAGGTGAAA	
klf1_R	ACTCATACGGCTTCTGTCCG	
nampt-b_ F	ACAGACTCTTATAAGATTACCCACTAC	NM_212668.2
nampt-b_ R	GGCCATCATATTTCTCTAAAACTTTCC	

Supplementary Table 2. An example of results from Dunn's multiple comparison test. *nampt-a*$^{+/+}$ (+/+) and *nampt-a*$^{t10pm/t10pm}$ (-/-) embryos were injected with *nampt-a* morpholino (MO). For the statistical analysis of the fold change in target gene expression normalized to *actb1* expression between different treatments, the *p*-value and *z*-statistics were quantified using Prism (GraphPad Software). The values presented are for *kdrl* expression (Fig. 4A) with the total number of values as 54 (15 + 12 + 15 + 12 samples in the different conditions).

**Dunn's multiple comparison test**	**Adjusted *p*-value**	***z***
+/+ compared to +/+ with MO	0.0005	3.943
+/+ compared to -/-	0.0007	3.859
+/+ compared to -/- with MO	0.0159	3.007
-/- compared to -/- + MO	> 0.9999	0.6319

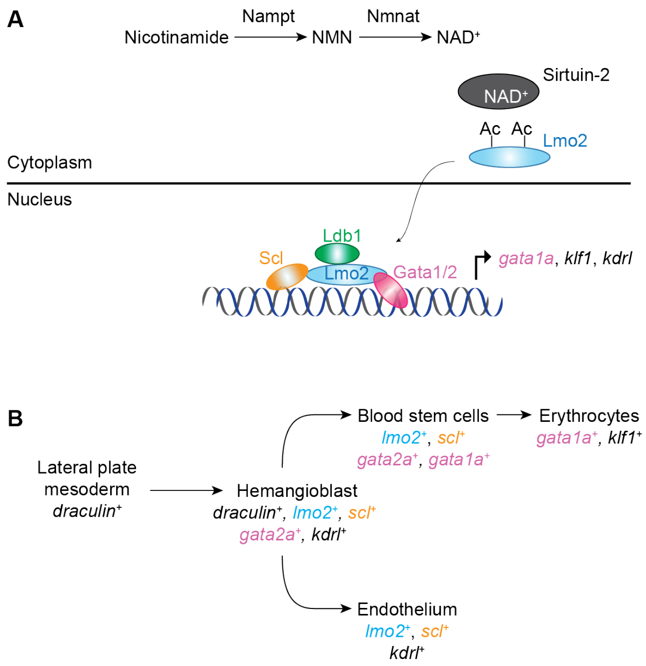

Supplementary Figure 1 | Key steps in the NAD$^+$ salvage pathway and primitive hematopoiesis. (A) The NAD salvage pathway recycles NAD$^+$ as a cofactor for Sirtuin-2, which is thought to deacetylate Lmo2 for the assembly of a hematopoietic transcription factor complex. In the NAD$^+$ salvage pathway, NAD$^+$ levels in cells are restored by Nampt first converting nicotinamide to nicotinamide mononucleotide (NMN). NMN adenyltransferase (Nmnat) then converts NMN to NAD$^+$. Lmo2 acts as a scaffold for Ldb1, Scl and Gata1/2, which together form a transcription factor complex for blood stem cell specification by inducing the transcription of *gata1a*, *klf1* and *kdrl*. The diagram is based on the proposed mechanism by Morishima et al. (2019). Ac: Acetylation. **(B)** Simplified schematic of primitive hematopoiesis with corresponding gene markers starting from lateral plate mesoderm to differentiated blood cells (erythrocytes) and endothelial cells.

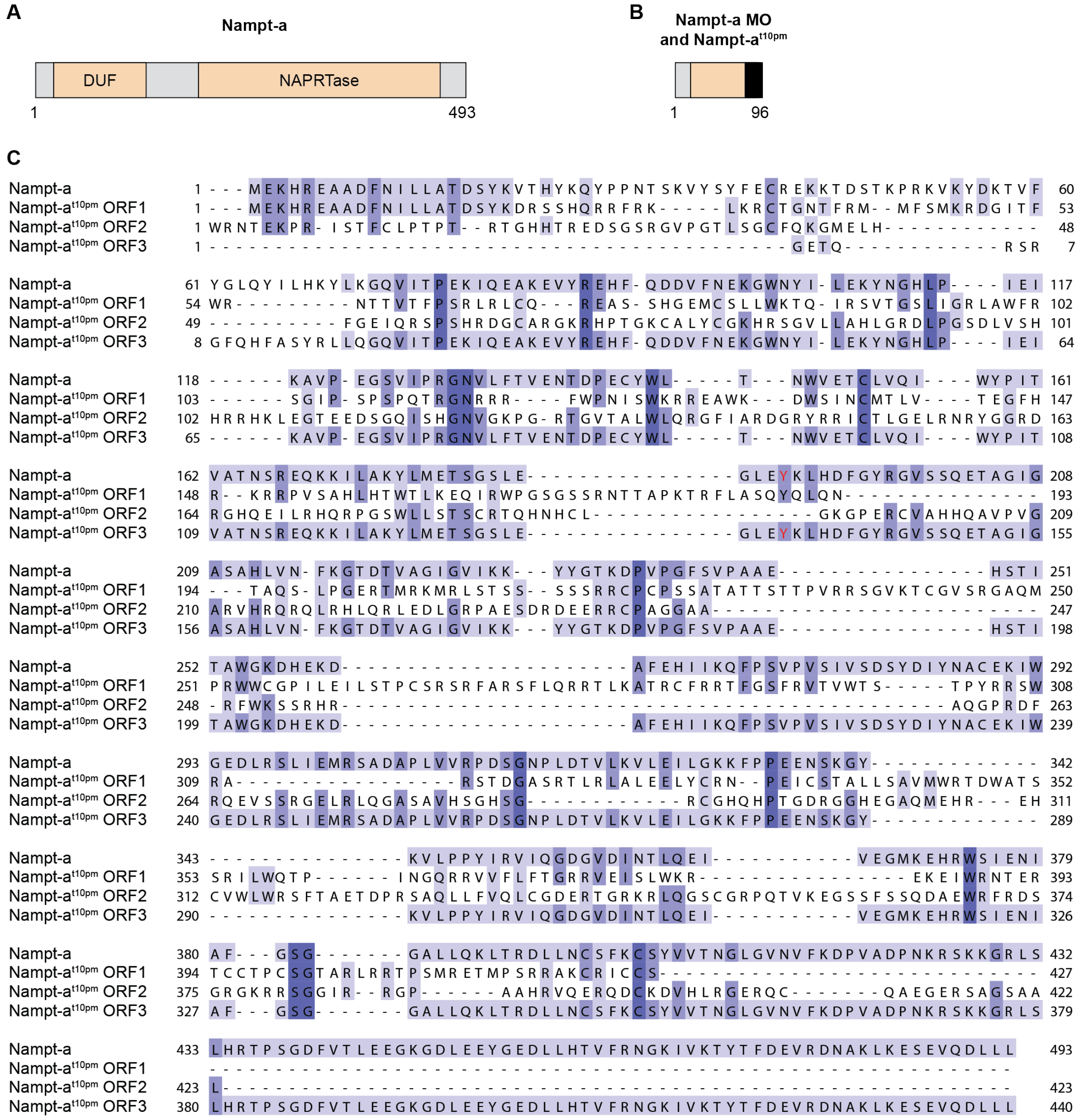

Supplementary Figure 2 | Possible Nampt-a^{t10pm} protein variants resulting from the translation of incorrectly spliced mRNAs. (A) Schematic of wild-type Nampt-a protein translated from the canonical translation start site. Wild-type Nampt-a contains an N-terminal domain of unknown function (DUF) and a C-terminal enzymatic domain of the Nicotinate phosphoribosyl transferase family (NAPRTase) starting at residue Y189 (marked red in (C)). **(B)** Schematic of mutant Nampt-a protein translated from the canonical translation start site. The protein product of morphants and *nampt-a^{t10pm}* mutants is expected to be truncated upstream of the NAPRTase domain. **(C)** Nampt-a^{t10pm} mutant proteins from three different frames were predicted using Expasy (Duvaud et al., 2021). These variants were aligned to the wild-type Nampt-a amino acid sequence using ClustalO in Jalview (Waterhouse et al., 2009). The color scheme represents percentage identity with white indicating no similarity, light blue indicating one third identical residues, medium blue indicating two thirds identical residues, and dark blue indicating 100% identical residues. The red Y marks the start of the NAPRTase domain. See Supplementary Note for further details.

Supplementary References

NCBI. (1988). National Center for Biotechnology Information. National Library of Medicine (US), *National Center for Biotechnology Information*. https://www.ncbi.nlm.nih.gov/

The UniProt Consortium. (2023). UniProt: the Universal Protein Knowledgebase in 2023. *Nucleic Acids Research*, 51(D1), D523-D531. https://doi.org/10.1093/nar/gkac1052
